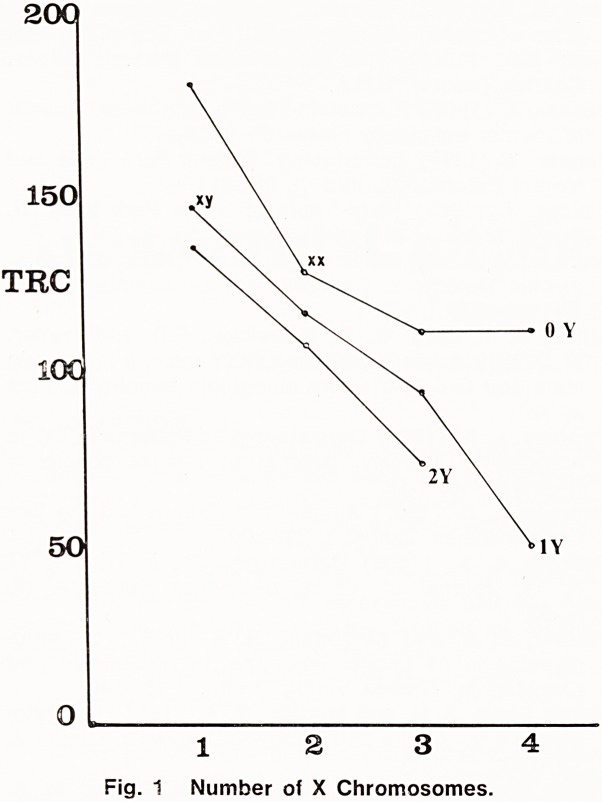# Dermatoglyphics in Medicine

**Published:** 1971-04

**Authors:** T. J. David

**Affiliations:** Bristol Royal Infirmary


### Dermatoglyphics in Medicine

**T. J. David, M.B., Ch.B.**
Bristol Royal Infirmary

### HISTORY

The first known observations of dermal ridges were made by Nehemiah Grew (1641-1712) who wrote in the Philosophical Transactions of 1684 "For if any one will but take the pains, with an indifferent Glass, to survey the Palm of his Hand very well washed with a Ball; he may perceive innumerable little Ridges, of equal bigness and distance, and everywhere running parallel one with another. And especially upon the ends and first Joynts of the Fingers and Thumb, upon the top of the Ball, and near the root of the Thumb a little above the Wrist. In all which places, they are very regularly disposed into spherical Triangles and Elliptics. Upon these Ridges stand the Pores, all in even Rows, and of that magnitude, as to be visible to a very good Eye without a Glass." Marcello Malpighi (1628-94). a contemporary of Grew, made some passing references to the papillary ridges arranging themselves as patterns. In 1823 John Purkinje (1787-1869) submitted a thesis on fingerprint classification, but little notice was taken of his work at the time. It was left to three great Englishmen, Sir Francis Galton (1822-1911), Sir William Herschel (1833-1917) and Sir Edward Henry (1859-1931) to produce the fingerprint system now used throughout the world. By 1901, finger print identification was practised in England, replacing Bertillon's anthropometric methods of personal identification.

### INTRODUCTION

The word "dermatoglyphics" which literally means the patterns formed by the epidermal ridges of the skin (derma, skin + glyphe, carve), was given a second meaning by Professor Harold Cummins in 1926 who used the word to describe the study of these patterns on the fingers, palms, soles and toes of humans as well as certain higher primates. Strictly speaking, dermatoglyphics does not include the study of palmar creases, although these may be relevant in their own right (e.g. 30 to 40% of patients with Down's syndrome have a single transverse palmar crease). Dermatoglyphics have been studied in various diseases since the end of the last century, but the failure of the subject to arouse wide interest is mainly due to the extremely poor standard of most communications on this important subject. This is partly because many published studies have dealt with very insignificant numbers of cases, and partly because of a general lack of attention to the technical side of the subject by doctors who mave had little experience of the taking or interpretation of finger and palm prints. It is also due to the present "disease approach" to dermatoglyphics; that is the study of dermatoglyphics in one particular disease. So far the most important findings have been made by a "dermatoglyphic approach"; that is the study of a single dermatoglyphic parameter in different diseases, and a notable recent example is the result of the Total Ridge Count (see below) in anomalies of the sex chromosome complement.

The permanence of fingerprints throughout life was first established by Sir Francis Galton (Galton, 1892). Although it is not known exactly when fingerprint patterns are formed during intra-uterine life, certain intra-uterine growth disturbances affecting the extremities (e.g. thalidomide phocomelia, dominant ectrodactyly, and ectrodactyly due to hypoxia) will be accompanied by abnormal dermatoglyphics (Jancar, 1967).

The skin of human palms and soles possesses easily visible epidermal ridges. These are the site of sweat pore openings as well as of sensory nerve endings. In small areas these ridges appear to run in parallel lines, and where three such areas meet a "triradius" is said to be formed (Plate I). The triradius is an

**Plate I. Triradius.**

important feature for the classification of fingerprint patterns; in this connection it is known to the police as a "delta", which has minor important differences from a triradius since it implies the presence and enclosure of a definite pattern whereas a triradius does not imply the presence of a pattern. On palms triradii form the main dermatoglyphic landmarks, and are not necessarily associated with a pattern. The triradius is a biological phenomenon not confined to epidermal ridges, and a good example of a triradius in nature is the triradius formed by the stripes of a zebra (Plate II).

**Plate II.**

**FINGERPRINTS**

It is conventional to classify fingerprints into different patterns, and these are described below with correct abbreviations in parentheses.

(a) **Arch** (A) (Plate III). The ridges run across the fingertip from one side to the other without forming a true pattern. There is no triradius.

**Plate III. Arch.**

**Plate IV. Tented Arch.**

(b) **Tented Arch** (T) (Plate IV). This is a variation of the arch. The ridges still run across the fingertip from one side to the other, but near the centre of the pattern the ridges are held up by a vertical ridge which takes its origin from or near the triradius. The pattern looks like the outline of a tent — hence the name "tented arch".

**Plate V. Loop.**

(c) **Loop** (Plate V). In the loop there are some ridges which re-curve through 180 degrees like a hairpin. In association with a loop there is always a triradius. Loops either open out onto the ulnar or radial border of the finger, and are classified as ulnar (U) or radial (R) loops respectively.

Plate VI. Twinned Loop.

Plate VII. Whorl.

(d) **Twinned Loop** (TL) (Plate VI). The twinned loop possesses two distinct loops which embrace one another, and one loop is the ascending loop while the other is the descending loop. There are two triradii, and for a pattern with two loops to be a twinned loop the two triradii must, by definition, be on *opposite* sides of the ascending loop.

(e) **Whorl** (W) (Plate VII). The ridges run in a more or less concentric circular direction, and there are two triradii.

(f) **Lateral Pocket Loop** (LP) (Plate VIII). Usually known as a "lateral pocket", this pattern has two loops; but unlike the twinned loop both triradii must, by definition, be on the *same* side of the ascending loop.

(g) **Composite** (Comp) (Plates IX and X). There are many different types of composites, but in general they do not conform to any of the patterns above, and usually have more than two triradii.

Ulnar loops are by far the commonest fingerprint patterns, forming about 60 to 70% of all fingerprints. Next come whorls, twinned loops, radial loops, arches and tented arches. Lateral pockets and composites are distinctly rare, but should not be classified as whorls as they have a significance of their own (David 1969, 1970).

Plate VIII. Lateral Pocket Loop.

Plate IX. Composite.

Plate X. Composite.

### PALM PRINTS

A normal palm print is shown in Plate XI. At the base of each finger there is a triradius, and each one is labelled, from "a" under the index finger to "d" under the little finger. There is another triradius near the wrist between the thenar and hypothenar eminences,

Plate XI. Normal palm print, right palm.

and this is called the palmar "axial" or "t" triradius. Patterns are also found on the palm, mainly in three special sites: (a) on the thenar eminence, where they tend to be vestigial patterns rather than true loops or whorls, (b) on the hypothenar eminence, and (c) on interdigital spaces; by convention these are indicated in Roman numerals, from I (space between base of thumb and base of index finger) to IV (space between bases of ring and little fingers).

### TOE PRINTS

Toes possess the same types of patterns as fingers, although arches are commoner and whorls are fewer on the toes. An ulnar loop on a finger corresponds to a "fibular" loop on a toe, and a radial loop on a finger corresponds to a "tibial" loop on a toe.

### SOLE PRINTS

A normal sole print is shown in Plate XII. Over most of the sole the ridges tend to run transversely without forming a pattern, and the main pattern bearing area is on the ball of the foot, called the "hallucal" area.

### METHODS

Prints of ridged skin are usually very easy to obtain, except in small infants and some un-cooperative severely subnormal patients. Special fingerprint ink is rolled into a very thin film on a copper plate with a rubber roller. The fingers are then rolled, first on the copper plate, and then on to forms specially designed for fingerprinting. The finger is carefully controlled by the operator, care being taken to remove perspiration first with ether, and to avoid excessive pressure when

**Plate XII. Normal sole print, left sole.**

**Plate XIII. Right sole print of patient with Down's Syndrome, showing malformed ridges over most of the sole.**

making the print. It is good dermatoglyphic practice to ensure that one obtains two sets of fingerprints. One is taken as above. The other set of "plain" fingerprints is taken by placing both thumbs together on the ink and then the paper, followed by placing all eight fingers on to the ink and then the paper. This is a way of making sure that each rolled fingerprint is correctly identified with the appropriate finger. Palm prints are recorded by rolling the ink on to the palm with a rubber roller, and then pressing the palm on to a piece of plain white paper, preferably with a piece of rubber or felt underneath the paper so that the hollow of the palm appears on the print. Foot prints are less easy to record, partly because the ridged skin extends up the sides of the foot, and sole and toe prints require more time and experience. Prints taken in this way are a mirror image of the epidermal patterns, and the ridges appear black with the sweat pores visible as white dots.

Other methods, which are not as satisfactory as the one above, include the use of a special invisible ink and special pre-treated paper, and the use of rubber or plaster casts of the hands or feet.

### DERMATOGLYPHICS IN DISEASE

Many diseases have been studied so far, and the important findings are outlined below.

1. **Down's Syndrome** (G-Trisomy)

There is a tendency for there to be fewer whorls, arches, and radial loops than normal, with an increase in the number of ulnar loops on the fingers. There are two classical appearances in Down's syndrome. In one all ten fingerprints are ulnar loops. In the other the fingerprints are ulnar loops, except for one or both ring fingers which bear radial loops. A radial loop on the ring finger is a distinctive feature in some patients with Down's syndrome, because if there are only one or two radial loops in a set of fingerprints then they are almost invariably on the index finger and only rarely on the ring finger. Striking features on the palm include (a) a large hypothenar pattern, found in 85% of mongols but in only 12% of controls (Ford Walker, 1957), associated with a triradius which, because the pattern is large, is situated near the centre of the palm, and is often mistakenly taken to be the triradius which in fact is still present near the wrist, (b) either no thenar pattern at all or a very small one, and (c) a third interdigital (III) loop. Another classical feature is the absence of any pattern on the hallucal area of the sole. Such a pattern is absent in about one half of all mongols, but it is only rarely absent in normal people (Holt, 1968). In addition, the ridges themselves may be malformed and appear as multiple dots (Plate XIII). Extreme examples of this have been described by Wolf, Brehme, Baitsch and Reinwein (1964).

Dermatoglyphics can in fact be used to diagnose Down's syndrome, and Ford Walker (1957) has devised a discriminant index as a "purely objective" diagnostic method. However, the author feels that Down's syndrome is a clinical diagnosis, and that the place of dermatoglyphics is as a collection of physical signs rather than as a "special investigation". In most instances the diagnosis of Down's syndrome is fairly simple, and does not require confirmation by karyotyping, although this may still be justifiable for genetic counselling. However there are some doubtful cases in which it is difficult to be sure, mainly in neonates in whom the diagnosis may present problems. Here dermatoglyphics can be very helpful, but must not be used as a substitute for either a careful physical examination or a critical examination of the chromosomes.

2. **Patau's Syndrome** ($D_1$ Trisomy)

Ulnar loops and whorls are less frequent on the fingers in $D_1$ trisomy than in controls, but radial loops and arches are more frequent than in controls (Penrose, 1966). In the palms, the striking features are a very distally displaced t triradius (Plate XIV), as well as the frequent presence of thenar patterns. The distal displacement of the t triradius can be measured by taking the atd angle, which should in theory increase as the t triradius becomes more distal in position. In practice the atd angle should not be quoted without a statement that other factors which also affect the atd angle are not in operation (adduction or abduction of the fingers when taking the print, width and breadth of the palm, age of subject, displacement of the a or d triradius, pressure used in taking the print, and lateral deviation of the t triradius). A IIIrd interdigital loop is usually present, as in Down's syndrome.

Dermatoglyphics may be particularly useful in $D_1$ trisomy because such infants are often stillborn or die before cytogenetic investigation can be made, and the dematoglyphic findings are usually even more striking than in Down's syndrome.

3. **Edward's Syndrome** (E Trisomy)

The very striking feature in E trisomy is the enormous preponderance of arches on the fingers, where they form 86.9% of all patterns in patients compared with 5% of patterns in controls (Penrose, 1969),

**Plate XIV. Left palm print, showing distal displacement of the t triradius.**

coupled with a reduction in the frequency of all other patterns. In the palms, the t triradius may be slightly displaced distally, but not nearly so much as in $D_1$ trisomy.

4. **Sex Chromosome Abnormalities**

The small differences that have been observed in the frequencies of fingerprint patterns compared with controls appear to be related to changes in the Total Ridge Count (TRC). The ridges crossing a straight line between the core of the pattern and the triradius are counted. For an arch or tented arch the ridge count is 0, and for a twinned loop, lateral pocket, or whorl, only the larger of the two possible counts is taken. The ridge counts on all ten fingers are summated to produce the TRC. The TRC appears to have a linear relationship with both the number of X chromosomes and the number of Y chromosomes, although the former appears to have about three times more effect on the TRC than the latter (Penrose, 1967). (See Fig 1).

There are several possible explanations for this linear relationship between the number of sex chromosomes and the TRC, the most likely and least contrived one being that the TRC is polygenically inherited (for which there is already evidence (Holt, 1952), and that of the genes for TRC some are located on the sex chromosomes. Another possible explanation is that the presence of extra sex chromatin, since it appears to replicate late in cell division, may delay cell division slightly and this may in some way allow the TRC to increase.

Most palmar changes found in sex chromosome abnormalities are non-specific, except that the d triradius has been found to be absent in 3 out of 4

Fig. 1 Number of X Chromosomes.

patients with XXXXY constitution in Bristol, as well as in another published case (Miller et al, 1961). This is a very rare finding in normal people.

5. **Schizophrenia**
A considerable amount of work has been done in this field, and not all the findings are in agreement with one another. This disparity may stem from variations in diagnostic criteria, geographical variations, and variations in dermatoglyphic criteria. Recent work has shown that childhood schizophrenics have an exaggeration of the normal inter-sex differences, whereas adult schizophrenics appear to have a "levelling" of sex differences (Sank, 1968).

6. **Rubella Embryopathy**
The finding of an increased incidence of whorls in congenital rubella (Purvis Smith et al, 1968) has now been confirmed (Purvis Smith et al, 1969). In addition an abnormal single transverse palmar crease has been described.

7. **Wilson's Disease**
Hodges and Simon (1960) found that 20 selected patients with Wilson's disease had a significantly increased number of whorls on certain fingers. This has never been confirmed, and 6 such patients studied in Bristol so far do not show this trend, although this is an inadequate number to base any firm conclusions upon. It would seem worthwhile to study not only patients with Wilson's disease, but also their parents and children, since the disease is likely to be inherited as a Mendelian recessive character, and it would be helpful to be able to detect clinically normal heterozygous carriers by taking their fingerprints.

8. **Huntington's Chorea**
The possibility of detecting heterozygotes with this disease by dermatoglyphics has been explored, and it has not been found to be possible, although a slight increase in whorls was found (Barbeau et al, 1966). Huntington's chorea is inherited as a Mendelian dominant character, but the average age of onset is about 35 years, usually after an affected person has transmitted the gene to half his children.

9. **Leukaemia**
Since the report of abnormal palmar creases in leukaemia by Menser and Purvis Smith (1969), much correspondence has appeared on this subject. To find that a single transverse palmar crease is a feature of leukaemia would be to add a third link between Down's syndrome and leukaemia, the other two being the high risk of patients with Down's syndrome dying of leukaemia, and the finding of an abnormal G chromosome ("Philadelphia chromosome") in some patients with leukaemia. However, despite a great deal of work in this field, the finding of abnormal creases in leukaemia is not in general confirmed (Verbov, 1970a). Whether small changes in pattern frequency will be confirmed remains to be seen, but this would seem to be unlikely.

10. **Congenital Heart Disease**
Sanchez Cascos (1964) suggested that one could use fingerprints to diagnose pulmonary stenosis, aortic stenosis, coarcation of the aorta, Fallot's tetralogy and ventricular septal defect. Recently it has been put forward (Verbov, 1970b) that palm prints might be used "as a guide to distinguish between congenital and acquired heart disease in patients with cardiac murmurs of unknown etiology". A careful study of over 300 patients and their families in Bristol lends no support to either of these truly remarkable claims. The preliminary findings (David, 1969) suggested (a) that fingerprints may be a useful indication that the cardiac defects detected in certain children are multiple, and (b) that it may be possible to detect a familial factor in certain cases of congenital heart disease by examining the finger and palm prints, and this would be useful for giving more accurate genetic counselling to the parents and the affected patients themselves. The Bristol study is continuing.

11. **Coeliac Disease**
It has been found in Bristol that patients with untreated adult coeliac disease (and many treated ones as well) "suffer" not only from loss of their intestinal villi but also some loss of their fingerprints as well. The two changes appear to be correlated. In addition, it has been found that when patients are treated with a gluten free diet the clarity of their fingerprints appears to return to a certain extent, again in parallel with the partial return of intestinal villi. The change of epidermal ridge atrophy appears to be confined to adult coeliac disease, since many other wasting diseases have been studied with normal results (David et al, 1970).

12. **Other Diseases**
Many other diseases have been studied. These include phenylketonuria (Alter, 1967), Parkinson's disease (Barbeau et al, 1966), Cooley's anaemia (Rosner et al, 1969), diabetes mellitus (Chakravartti, 1967) alopecia areata and psoriasis (Verbov, 1968), Rubinstein-Taybi's syndrome (Jancar, 1965) and the de Lange syndrome (Smith, 1966; Pfeiffer et al, 1967). The exact relevance of dermatoglyphics in these diseases is not yet clear.

### CONCLUSIONS

Dermatogyphic analysis is particularly worthwhile when applied to patients suffering from congenital abnormalities and mental retardation. Palm and foot prints have been collected at Stoke Park Hospital in Bristol for the past decade (Jancar, 1969), and this is now done regularly on all patients admitted for assessment. The study of dermatoglyphics is still in its infancy, but is becoming an important investigation in all branches of medicine, and its possibilities for research are almost unlimited.

**Acknowledgements**

I am very grateful to Dr. A. Raper who helped in the preparation of the manuscript, to all the doctors who permitted me to fingerprint their patients, and to the many members of the staff of the United Bristol Hospitals who allowed me to use them as controls. I am indebted also to Dr. J. Jancar, Consultant Psychiatrist at Stoke Park Hospital, for his continued helpful criticism, to Dr. N. Royston and Dr. B. Webb, Consultant Paediatricians in Yeovil and Taunton, for their great help and interest, and to the S.W. Regional Cytogenetics Laboratory, Southmead Hospital, Bristol.

### REFERENCES

**Alter, M.** (1967) Dermatoglyphics in Phenylketonuria, Humangenetik **4,** 23-28.

**Barbeau, A., Trudeau, J-G., and Coiteux, C.** (1965) Fingerprint Patterns in Huntington's Chorea and Parkinson's Disease, Canadian Medical Association Journal. **92,** 514-516.

**Chakravartti, M. R.** (1967) Assocation between Diabetes mellitus and Dermatoglyphics, in Hautleisten und Krankheiten, Grosse Verlag Berlin, p.157--160.

**David, T. J.** (1969) Fingerprints in Congenital Heart Disease, Bristol Medico-Chirurgical Journal **84,** 167-169.

**David, T. J.** (1970) in Fingerprint and Identification Magazine, January.

**David, T. J., Ajdukiewicz, A.B. and Read, A. E.** (1970) Fingerprint Changes in Coeliac Disease, British Medical Journal **4,** 594-6.

**Ford Walker, N.** (1958) The Use of Dermal Configurations in the Diagnosis of Mongolism, Pediatric Clinics of N. America, May, 531-543.

**Galton, F.** (1892) Finger Prints, reprinted 1965 Da Capo, New York.

**Hodges, R. E. and Simon, J. R.** (1962) Relationship between fingerprint patterns and Wilson's disease, Journal of Laboratory and Clinical Medicine **60,** 629-640.

**Holt, S. B.** (1952) Genetics of Dermal Ridges: inheritance of total finger ridge count. Ann. Eugen. 17, 140.

**Holt, S.B.** (1968) The Genetics of Dermal Ridges, Charles Thomas, U.S.A.

**Jancar, J.** (1965) Rubinstein-Taybi's Syndrome. Journal of mental deficiency Research **9,** 265-270.

**Jancar, J.** (1967) Ectrodactyly, Spastic Paraplegia and Mental Retardation. ibid. II, 207-211.

**Jancar, J.** (1969) Sixty Years of Stoke Park Hospital. Bristol Medico-Chirurgical Journal **84,** 77-96.

**Menser, M. A. and Purvis Smith, S. G.** (1969) Dermatoglyphic Defects in Children with Leukaemia, Lancet **1,** 1076-1078.

**Miller, O. J., Breg, W. R., Schmickel, R.D. and Tretter, W.** (1961) A family with an XXXXY male, a leukaemic male and two 21-trisomic mongoloid females. Lancet **2,** 78.

**Penrose, L. S.** (1966) Dermatoglyphic Patterns in Large Acrocentric Trisomy. Journal of mental deficiency Research **10,** 1-18.

**Penrose, L. S.** (1967) Finger-Print Pattern and the Sex Chromosomes. Lancet **1,** 298-300.

**Penrose, L. S.,** (1969) Dermatoglyhpics in Trisomy 17 or 18. Journal of mental deficiency Research **13,** 44-59.

**Pfeiffer, R. A. and Kumbnani, H. K.** (1967) Dermatoglyphics in de Lange Syndrome, in Hautleisten und Krankheiten, Grosse Verlag Berlin, p.137-140.

**Purvis Smith, S. G. and Menser, M. A.** (1968) Dermatoglyphics in Adults and Congenital Rubella. Lancet **2,** 141-143.

**Purvis Smith, S. G., Howard, P. R. and Menser, M. A.** (1969) Dermatoglyphic Defects and Rubella Teratogenesis, Journal of American Medical Association, **209,** 1865-1868.

**Rosner, F. and Spriggs, H. A.** (1969) Dermatoglyphic Studies in Patients with Cooley's Anaemia. Annals of New York Academy of Science **165,** 378-386.

**Sanchez Cascos, A.** (1964) Finger-Print Patterns in Congenital Heart Disease. British Heart Journal **26,** 524-527.

**Sank, D.** (1968) Dermatoglyphics of Childhood Schizophrenia. Acta Genetica **18,** 300-314.

**Smith, G. F.** (1966) A Study of the Dermatoglyphs in the De Lange Syndrome. Journal of mental deficiency Research **10,** 241-254.

**Verbov, J.** (1968) Dermatoglyphic and Other Findings in Alopecia Areata and Psoriasis. British Journal of Cliincal Practice **22,** 257-259.

**Verbov, J.** (1970a) Dermatoglyphs in Leukaemia. Journal of medical Genetics **7,** 125-131.

**Verbov, J.** (1970b) Clinical Significance and Genetics of Epidermal Ridges — A Review of Dermatoglyphics. Journal of Investigative Dermatology **54,** 261-271.

**Wolf, U., Brehme, H., Baitsch, H., Kunzer, W. and Reinwein, H.** (1963) Lancet **2,** 887.

## Figures and Tables

**Plate I. f1:**
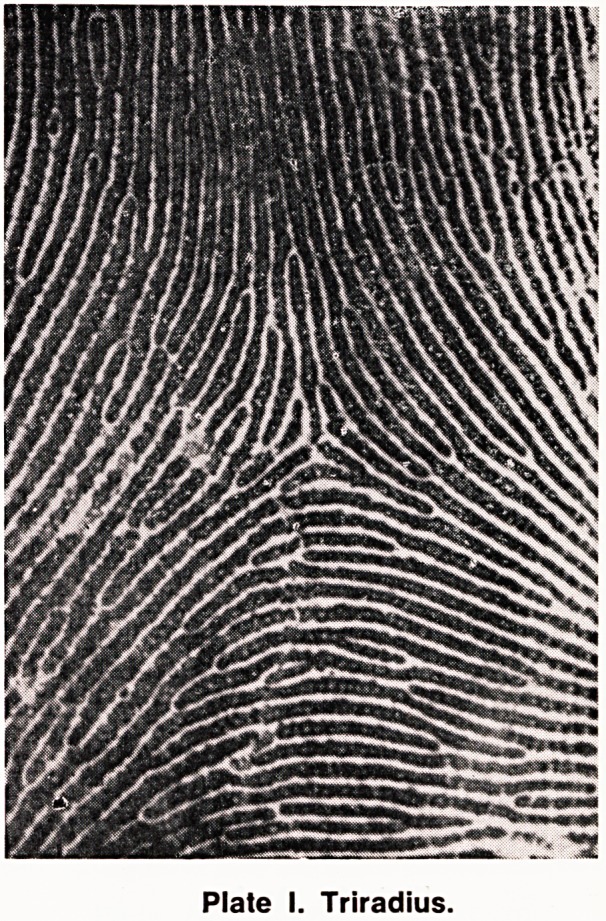


**Plate II. f2:**
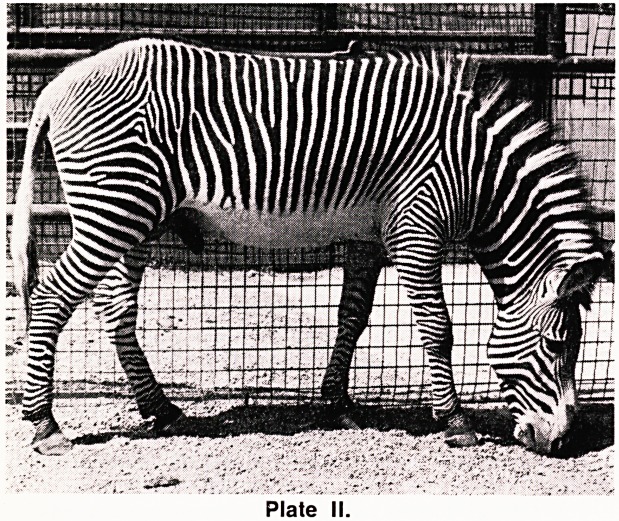


**Plate III. f3:**
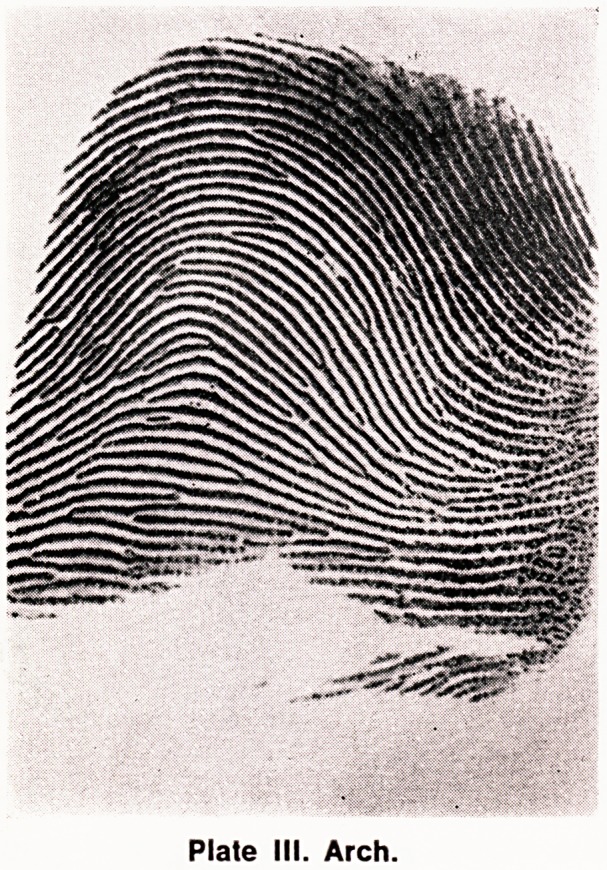


**Plate IV. f4:**
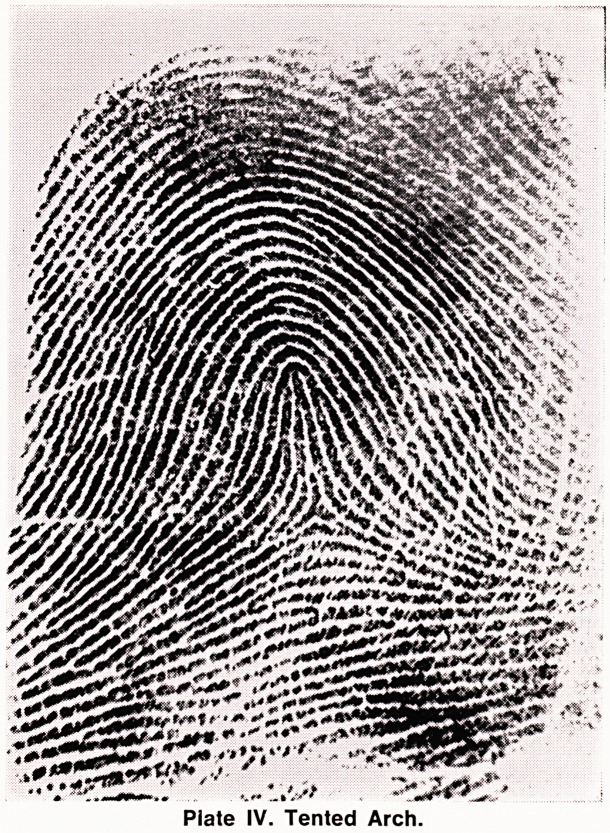


**Plate V. f5:**
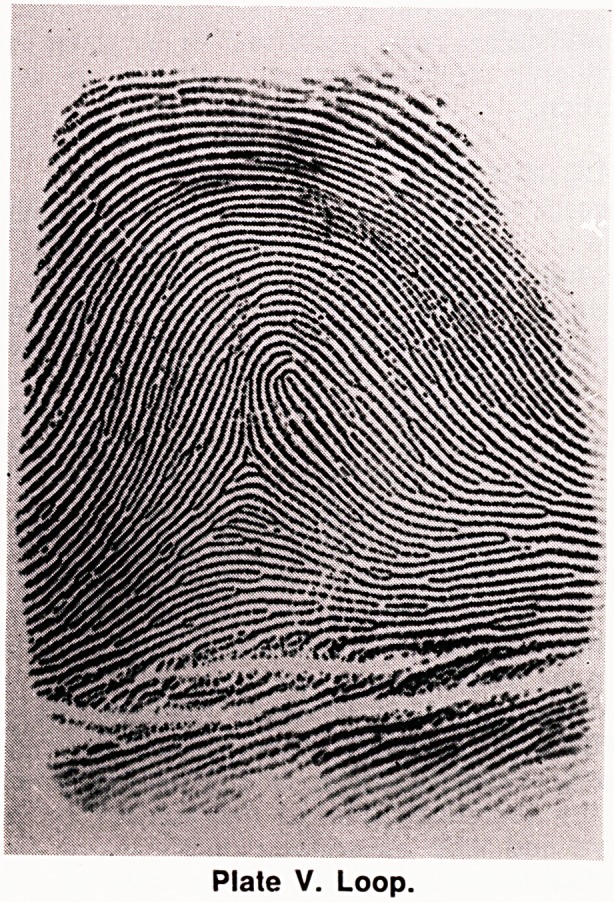


**Plate VI. f6:**
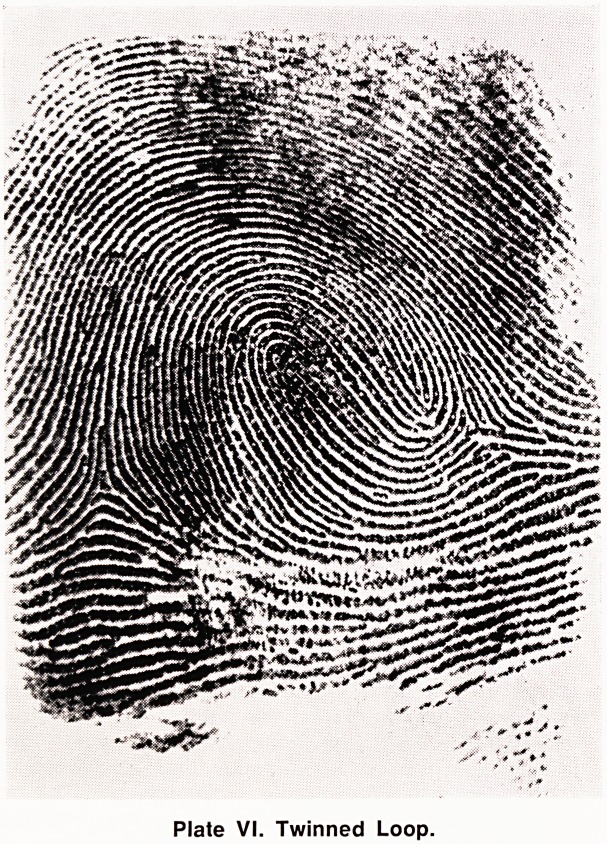


**Plate VII. f7:**
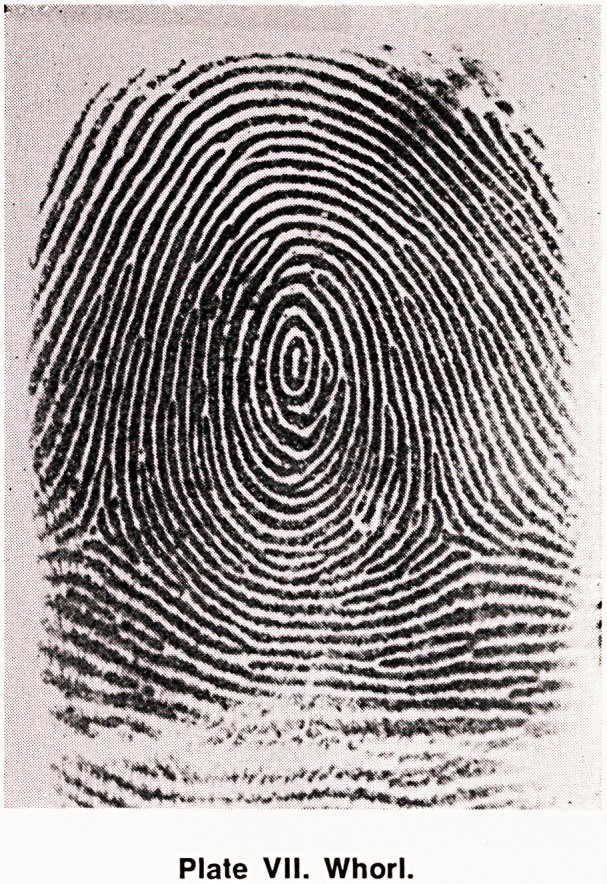


**Plate VIII. f8:**
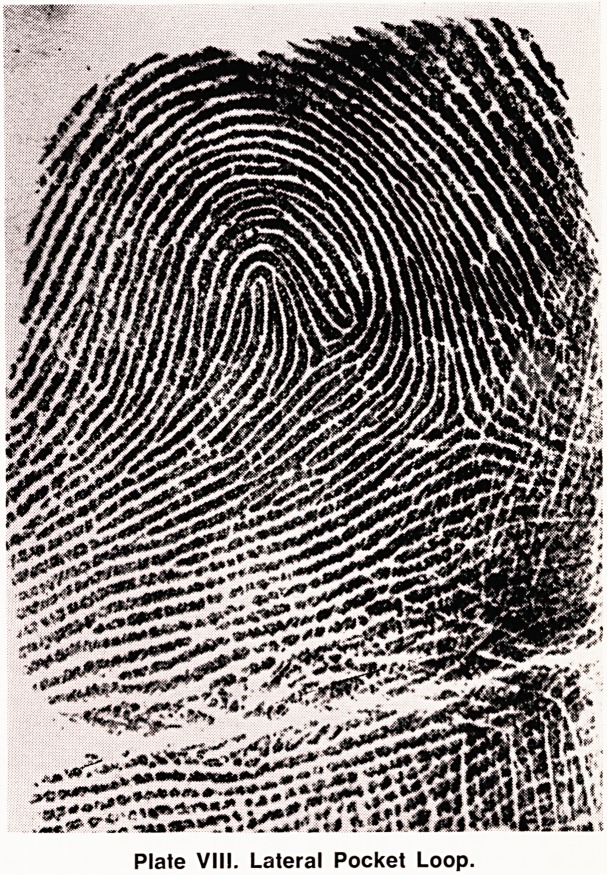


**Plate IX. f9:**
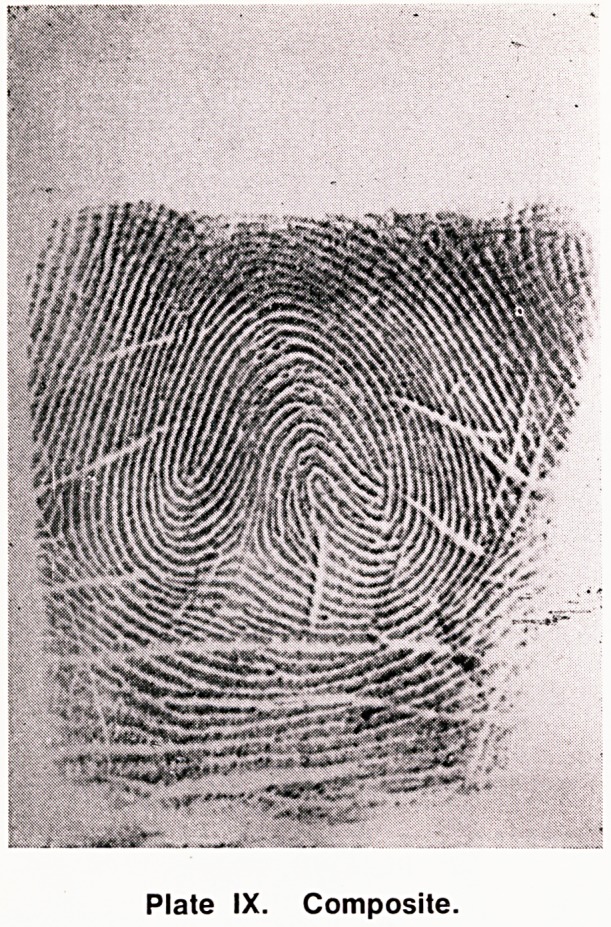


**Plate X. f10:**
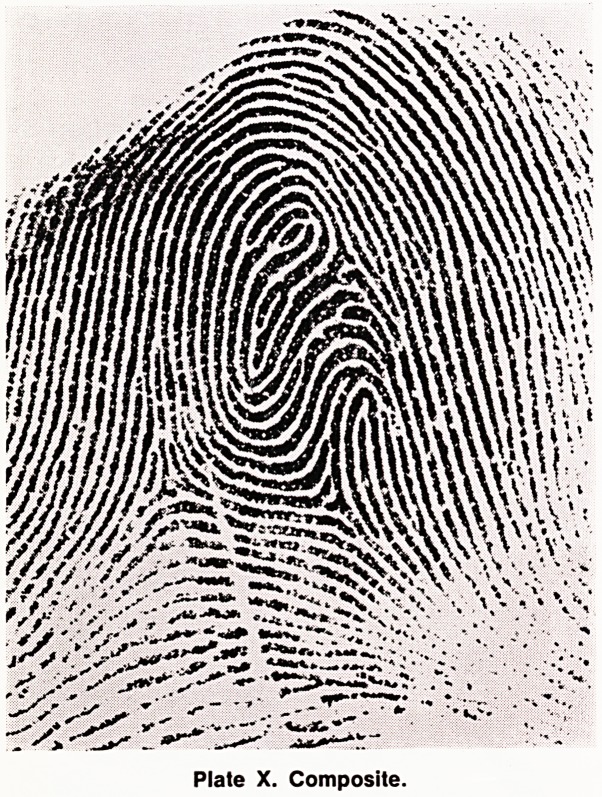


**Plate XI. f11:**
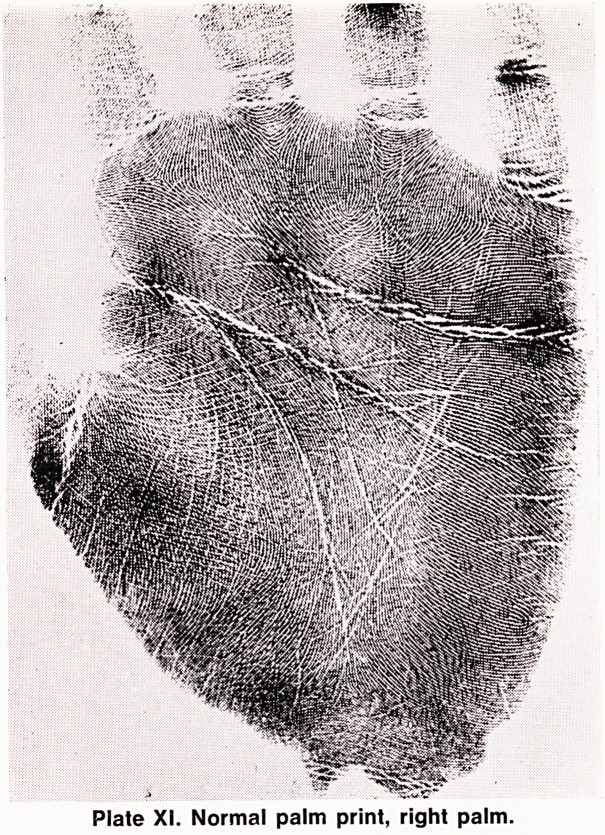


**Plate XII. f12:**
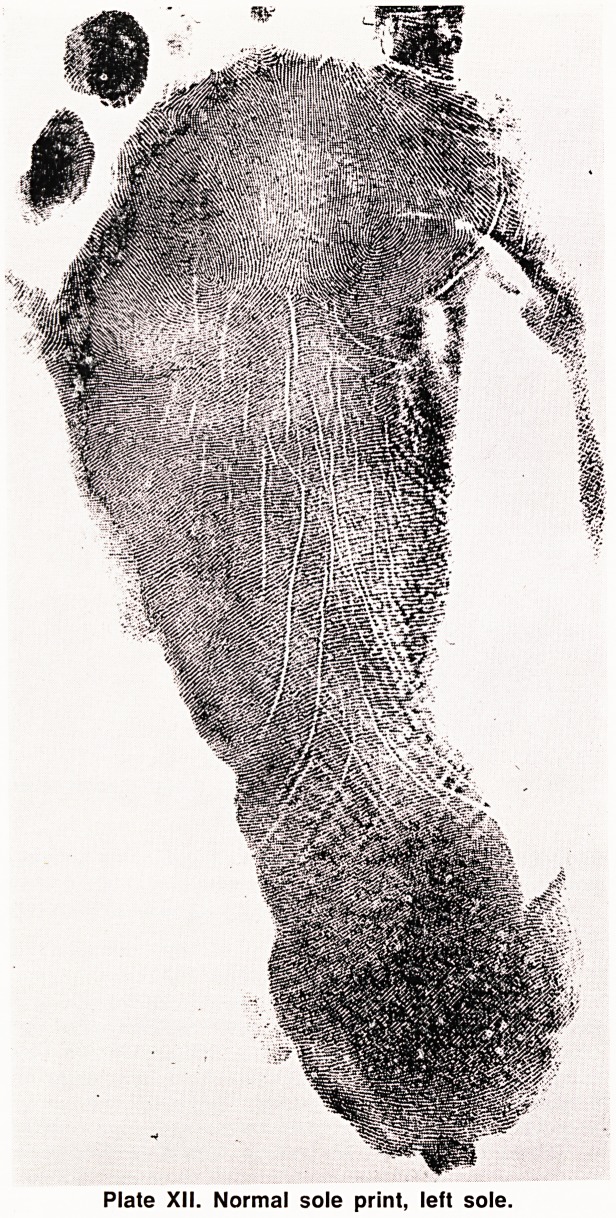


**Plate XIII. f13:**
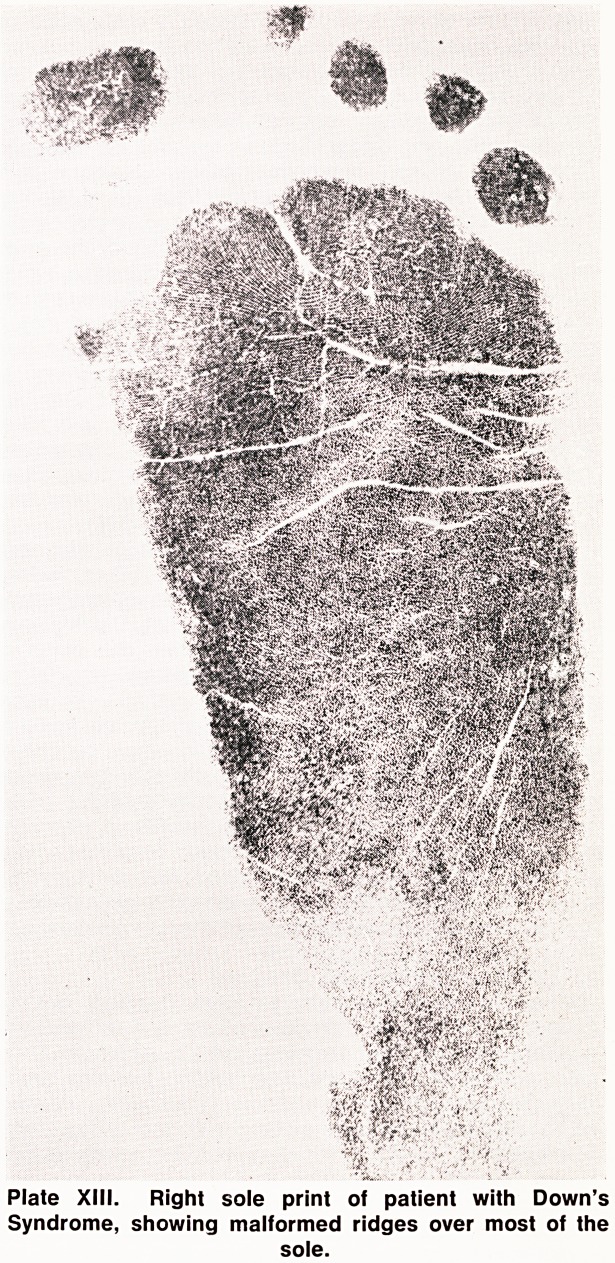


**Plate XIV. f14:**
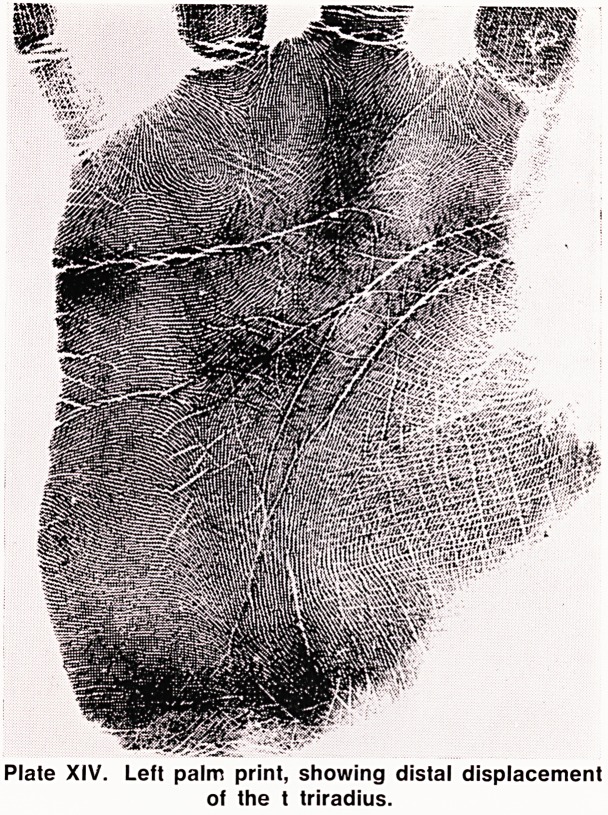


**Fig. 1 f15:**